# Development of a Derivatization Reagent with a 2-Nitrophenylsulfonyl Moiety for UHPLC-HRMS/MS and Its Application to Detect Amino Acids Including Taurine

**DOI:** 10.3390/molecules26123498

**Published:** 2021-06-08

**Authors:** Shusuke Uekusa, Mayu Onozato, Tatsuya Sakamoto, Maho Umino, Hideaki Ichiba, Kenji Okoshi, Takeshi Fukushima

**Affiliations:** 1 Department of Analytical Chemistry, Faculty of Pharmaceutical Sciences, Toho University, Chiba 274-8510, Japan; shuusuke.uekusa@phar.toho-u.ac.jp (S.U.); mayu.onozato@phar.toho-u.ac.jp (M.O.); tatsuya.sakamoto@phar.toho-u.ac.jp (T.S.); 3020001u@st.toho-u.jp (M.U.); ichiva@phar.toho-u.ac.jp (H.I.); 2 Department of Clinical Pharmacy, Faculty of Pharmaceutical Sciences, Toho University, Chiba 274-8510, Japan; 3 Department of Environmental Science, Faculty of Science, Toho University, Chiba 274-8510, Japan; kenji.okoshi@env.sci.toho-u.ac.jp

**Keywords:** taurine, glutamine, derivatization, clams, high-resolution mass spectrometry

## Abstract

Taurine (Tau) has some important ameliorating effects on human health and is present in bivalve. For the selective analysis of Tau with other amino acids, we designed a derivatization reagent, 2,5-dioxopyrrolidin-1-yl(4-(((2-nitrophenyl)sulfonyl)oxy)-6-(3-oxomorpholino)quinoline-2-carbonyl)pyrrolidine-3-carboxylate (Ns-MOK-β-Pro-OSu). After derivatization with Ns-MOK-β-Pro-OSu, amino acids with Tau in Japanese littleneck clams were determined through ultra-high-performance-liquid chromatography with high-resolution tandem mass spectrometry (UHPLC-HRMS/MS) using an octadecyl silica column. We could detect 18 amino acids within 10 min. Tau, valine, glutamine, glutamic acid, and arginine in the clams were determined in the negative ion mode using the characteristic fragment ion, C_6_H_4_N_1_O_5_S, which corresponded to the 2-nitrobenzenesulfonylate moiety. The fragment ion, C_6_H_4_N_1_O_5_S, was recognized as a common feature regardless of the amino acid to be derivatized, and it was convenient for detecting amino acid derivatives with high selectivity and sensitivity. Therefore, highly selective quantification using UHPLC-HRMS/MS was possible using Ns-MOK-β-Pro-OSu.

## 1. Introduction

Taurine (Tau) or 2-aminoethanesulfonic acid is an endogenous sulfur-containing compound that is produced from l-cysteine via hypotaurine. Tau acts as a neuroprotective agent in the central nervous system [1] and plays an important role in endogenous antioxidant activity, brain development, and retinal function [2]. In addition, Tau can be used for the biosynthesis of taurocholate, a bile acid important for digestive functions.

Tau deficiency is associated with the pathogenesis of some diseases [2,3]. Recently, we found a significant decrease in serum taurine levels in the prodromal stage of psychosis, an at-risk mental state (ARMS) through a metabolomic study [4]. However, it was difficult to separate amino acids without pre-column derivatization in an octadecyl silica (ODS) column because they were hardly retained.

Considering the physiological usefulness of Tau, the intake of Tau through food can also be important for maintaining healthy conditions. Tau is abundantly present in octopus, squid [5], and bivalve [6]. Among them, the Japanese littleneck clam, *Asari*, *Ruditapes philippinarum* is an important fishery resource edible bivalve usually cooked as an indispensable seafood dish in many countries. Some studies have examined the profiles of the amino acids present in the Japanese littlenecked clam in terms of their nutritional or taste aspects [7,8].

Previous studies analyzing amino acids in clam have used dansyl chloride [6] for derivatization, followed by separation using high-performance liquid chromatography (HPLC). Since recent years, high-resolution mass spectrometry (HRMS) is being widely utilized in environmental, biological, and pharmaceutical research [9,10].

**Figure 1:**
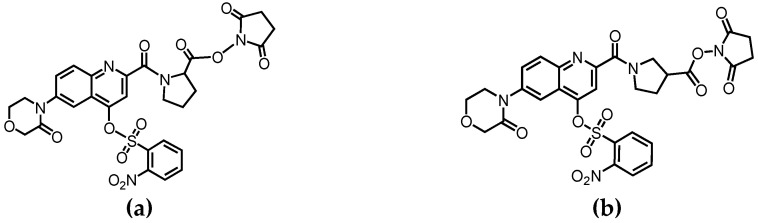
Chemical structure of Ns-MOK-Pro-OSu (**a**) and Ns-MOK-β-Pro-OSu (**b**).

Previously, we developed a diastereomer derivatization reagent, Ns-MOK-(*R*)- or -(*S*)-Pro-OSu (Figure 1a), which was used for derivatization with the amino group of an anti-epileptic drug, vigabatrin, which is a gamma-amino acid [11]. For ease of reaction with the amino group of the target drug, the phenolic hydroxyl group in the derivatization reagent was protected with a 2-nitrobenzenesulfonyl (nosyl) group. However, the reaction mixture was heated for more than 60 min to cause de-nosylation, releasing the nosyl group. This long heating time may be attributed to the steric hindrance of the reaction site with the derivatizing agent.

Thus, in the present study, we designed and developed a structural analog, Ns-MOK-β-Pro-OSu (Figure 1b), in which β-proline (β-Pro) was used instead of Pro. The activated ester moiety, 2,5-dioxopyrrolidin-1-yl, attached to β-Pro may react easily with the amino groups of analytes owing to less steric hindrance. The reagent was designed with less steric hindrance for enabling easy reaction with the amino groups.

The time-course profile showing the progress of the reaction with certain amino acids was examined and compared with that of Ns-MOK-β-Pro-OSu. The mass fragmentation patterns of some amino acids and Tau derivatized with Ns-MOK-β-Pro-OSu in collision cells were examined.

Finally, rapid analysis of the amino acid components including Tau in clams within 10 min was conducted using ultra-high-performance liquid chromatography (UHPLC)-HRMS/MS instrument equipped with an ODS column.

## 2. Results

### 2.1. Synthesis and Evaluation of 2,5-Dioxopyrrolidin-1-yl(4-(((2-nitrophenyl)sulfonyl)oxy)-6-(3-oxomorpholino)quinoline-2-carbonyl)pyrrolidine-3-carboxylate (Ns-MOK-β-Pro-OSu)

As in our previous reagent, Ns-MOK-Pro-OSu, the succinimide moiety was attached to the carboxyl group to produce an activated ester for labeling the primary amino group of the amino acid containing Tau. In addition, a 2-nitrobenzenesulfonyl (Ns-, nosyl) group was bound to a phenolic hydroxyl group at position 5 in quinoline ring to facilitate easy reactions of the amino group in the amino acid containing Tau. Comparing the retention times of Ns-MOK-Pro-OSu and Ns-MOK-β-Pro-OSu showed that Ns-MOK-(*S*)-β-Pro-OSu eluted before Ns-MOK-(*S*)-Pro-OSu from the ODS column (Figure S1), suggesting that rapid analysis using an ODS column is preferred for Ns-MOK-β-Pro-OSu.

### 2.2. Derivatization of Amino Acid with Ns-MOK-(R)- or -(S)-β-Pro-OSu

**Figure 2:**
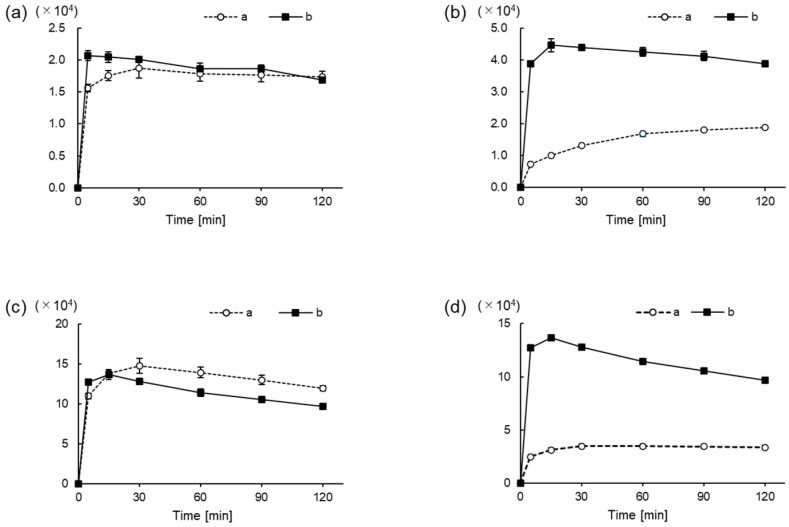
Time-course profiles of derivatization of amino acid: (**a**) Tau, (**b**) Glu, (**c**) Arg, and (**d**) Gln with Ns-MOK-(*S*)-Pro-OSu (dotted line with circles) and Ns-MOK-(*S*)-β-Pro-OSu (solid line with squares) by LC-TOF-MS.

Figure 2 shows time-course profiles of the derivatization reaction of the amino acid with Ns-MOK-β-Pro-OSu. According to the LC-TOF-MS data, which showed the [M + H]^+^ of the reaction product, the main products of the derivatization reaction were Ns-MOK-(*S*)-Pro- and (*S*)-β-Pro-amino acids. Regarding the reaction time, the peak area of the derivatives with Ns-MOK-(*S*)-β-Pro-OSu plateaued after 30 min. Conversely, in Ns-MOK-(*S*)-Pro-OSu, particularly in Glu, a longer reaction time was required to reach a plateau. Therefore, we decided to use Ns-MOK-(*S*)-β-Pro-OSu, which proved beneficial for reducing the reaction time and set the reaction time to 30 min.

### 2.3. Fragmentation of Amino Acid Derivatives with Ns-MOK-(S)-β-Pro-OSu

**Figure 3:**
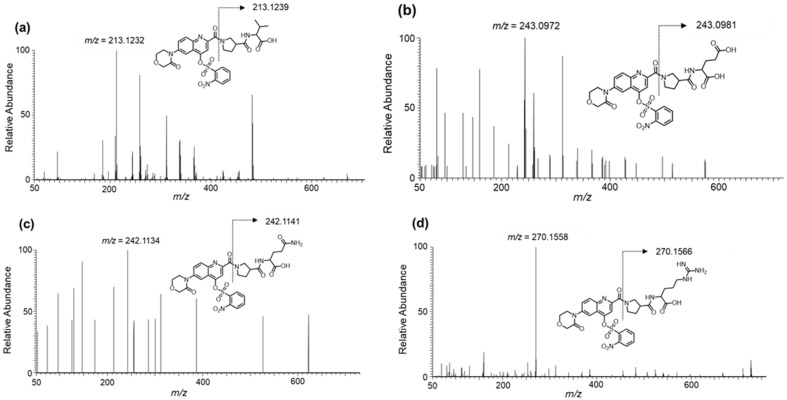
MS/MS spectra of derivatized amino acids: (**a**) Val, collision energy (CE) 30%; (**b**) Glu, CE 30%; (**c**) Gln, CE 30%; and (**d**) Arg, CE 30% with Ns-MOK-(*S*)-Pro-OSu using ESI (+) mode.

Figure 3 and Figure 5a show the MS/MS spectra of the fragment ions, namely Val, Glu, Gln, Arg, and Tau derivatives, obtained in the ESI (+) mode using UHPLC-HRMS. For all the amino acids, fragment ions cleaved at the β-Pro-attached amide bond were commonly observed under relatively small collision energies (CEs) of approximately 30 to 40%. The main fragments observed under each CE are given in Tables S1–S5.

**Figure 4:**
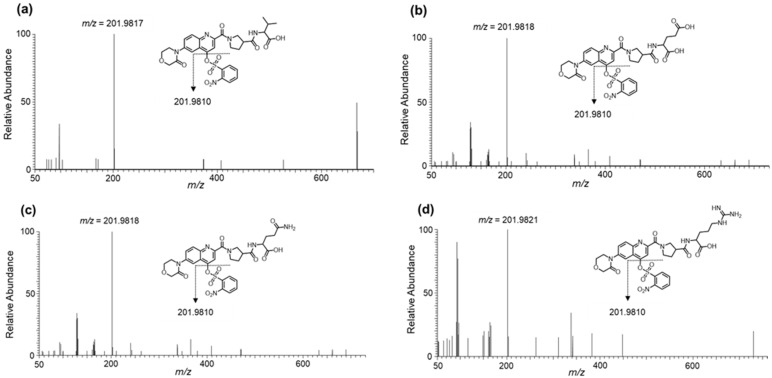
MS/MS spectra of derivatized amino acids: (**a**) Val, CE 20%; (**b**) Glu, CE 30%; (**c**) Gln, CE30%; and **(d**) Arg, CE 40%) with Ns-MOK-(*S*)-Pro-OSu using ESI (−) mode.

**Figure 5:**
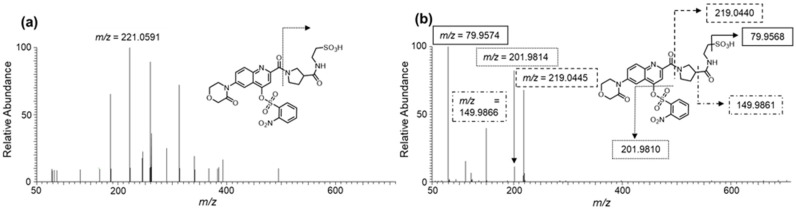
MS/MS spectra of derivatized Tau with Ns-MOK-(*S*)-Pro-OSu using ESI (+) CE 30% (**a**) and ESI (−) CE 30% modes (**b**).

Figure 4 and Figure 5b show the mass spectra of the fragment ions, Val, Glu, Gln, Arg, and Tau derivatives, obtained in the ESI (−) mode. Fragment ions cleaved at the nosylate moiety, C_6_H_4_NO_5_S, were commonly observed in all the amino acids. Furthermore, for Tau, in addition to this cleavage, fragment ions cleaved at the β-Pro-attached amide bond were observed. The main fragments in each CE are given in Tables S6–S10.

### 2.4. Detection of Amino Acids

**Figure 6:**
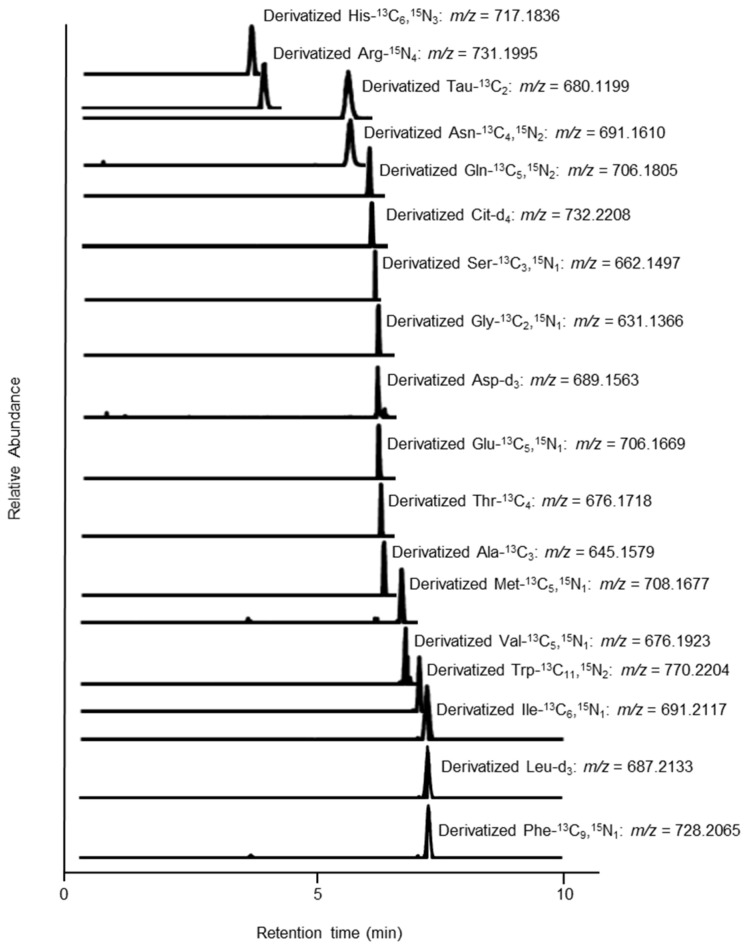
SIM chromatogram showing 18 stable isotope-labeled amino acid derivatives.

When the amino acid derivatives were separated by an ODS column and analyzed by UHPLC-HRMS, it was possible to detect 18 isotope-labeled amino acids using SIM mode within 10 min (Figure 6). The retention times of Leu and Ile differed by approximately 0.05 min, but these could not be separated.

### 2.5. Detection of Amino Acids in Bivalve

In our previous study, high levels of Glu and low levels of Tau were identified as risk factors in ARMS [4]. Therefore, we targeted bivalves containing a large amount of Tau. In addition to Tau and Glu, Gln and Arg, both of which are linked with Glu metabolism, and Val, which is abundant in bivalve, were quantified.

Calibration curves of the Val, Glu, Gln, Arg, and Tau derivatives obtained using the PRM mode are shown in Figure S7. Because the calibration curves showed R^2^ > 0.99, linearity was obtained in the tested concentration range. All the coefficients of variation in the calibration curve range were within 10% (Tables S11–S15). As shown in Table 1, the amino acids in the bivalve could be determined using the calibration curves. In the present method, the LOQs of Val, Glu, Gln, Arg, and Tau were in the range of 0.042–0.167 pmol. Tables S16–S20 present the quantification data for each amino acid (content per gram of the edible portion), indicating that differences in the amino acid contents existed among the bivalve.

**Figure 7:**
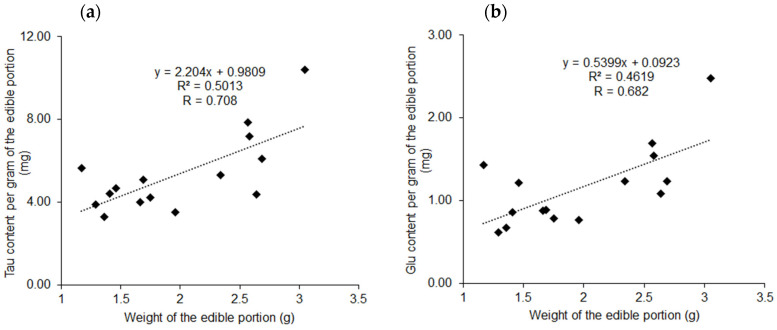
Scatter plot of the weight of the edible portion and Tau (**a**) or Glu (**b**) content per 1 g of the edible portion. Vertical axis: Tau (**a**) or Glu (**b**) content per gram of edible portion (mg); Horizontal axis: weight of the edible portion (g).

Positive correlations were found between the weight of the edible portion and the Tau and Glu contents per gram of the edible portion (*r* = 0.708, *p* = 0.00315 in Figure 7a and *r* = 0.682, *p* = 0.00514 in Figure 7b, respectively).

## 3. Discussion

For comparison with Ns-MOK-Pro-OSu, the derivatization reagent designed in our previous study [11], 2,5-dioxopyrrolidin-1-yl(4-(((2-nitrophenyl)sulfonyl)oxy)-6-(3-oxomorpholino)quinoline-2-carbonyl)pyrrolidine-3-carboxylate (Ns-MOK-β-Pro-OSu), a structural analogue, was synthesized.

Consequently, a time-course study revealed that Ns-MOK-β-Pro-OSu reacted with amino acids faster than Ns-MOK-Pro-OSu at room temperature. Furthermore, the peak area values of Glu and Gln were remarkably high when Ns-MOK-β-Pro-OSu was used (Figure 3). The reaction proceeded at room temperature with no release of the nosylate moiety in the produced derivative. Therefore, we could observe proper fragmentation of the nosylate moiety, C_6_H_4_N_1_O_5_S, in the MS/MS spectrum of the derivative with Ns-MOK-β-Pro-OSu.

In our previous study, Ns-MOK-Pro-OSu was reacted with amino groups under heating conditions to eliminate the nosyl group [11]. In contrast, Ns-MOK-β-Pro-OSu reacted effectively with amino acids at room temperature, and no nosyl elimination product was observed. This difference in the reaction with amino acids may be due to the lower steric hindrance of β-Pro-OSu than that of Pro-OSu. Thus, Ns-MOK-β-Pro-OSu was a more suitable reagent for MS/MS detection than Ns-MOK-Pro-OSu.

Generally, derivatization reagents for LC-MS/MS detection possess suitable structures for efficient fragmentation [12]. As mentioned above, Ns-MOK-β-Pro-OSu, exhibited unique fragmentations in the ESI (+) and ESI (−) modes, as observed in the MS/MS spectra (Figure 4, Figure 5 and Figure 6).

In addition, as Ns-MOK-(*S*)-β-Pro-OSu eluted earlier than Ns-MOK-(*S*)-Pro-OSu, we inferred that the analysis time could be shortened using Ns-MOK-(*S*)-β-Pro-OSu (Figure S1). Under the mobile phase conditions used in the present study, amino acids containing Tau could be analyzed within 10 min (Figure 7).

In the ESI (+) mode, cleavage occurred regularly at the β-Pro-attached amide bond; however, the signal-to-noise ratio of the ion fragment was not extremely high. In contrast, the nosylate moiety, C_6_H_4_N_1_O_5_S, was recognized as an intense fragment with a common structure regardless of the amino acid in the ESI (−) mode. The fragment ions of the nosylate moiety, C_6_H_4_N_1_O_5_S, were used for quantifying the amino acids. The NCE values set for each amino acid were between 20% and 40%, in which C_6_H_4_N_1_O_5_S was the most efficiently cleaved moiety. Therefore, this cleavage pattern could be used to detect the derivative with Ns-MOK-β-Pro-OSu with high selectivity and identify whether it was a derivative of Ns-MOK-β-Pro-OSu.

For detecting the Tau derivative, fragment ions cleaved at the SO_3_ moiety were observed. When searching for Tau on the *m/z* cloud (https://www.mzcloud.org/, accessed on 1 December 2020), SO_3_ fragment ions were observed from approximately 70 to 80% of the NCE. However, in the present study, fragment ions were observed under conditions where the NCE was relatively low. The SO_3_ fragment ions detected in MS^2^ at an NCE of approximately 30% may be useful to discriminate other compounds with sulfate groups.

Next, the present LC-HR-MS/MS technique using Ns-MOK-β-Pro-OSu was successfully applied to determine amino acids in the samples from the collected bivalve. As observed in Table 1, extremely high levels of Tau were found in the *R. philipinarum* compared to other amino acids. High levels of Tau in *R. philipinarum* have already been reported by Wang et al. [10], who also reported seasonal variations in the amino acid content of clams using LC-HR-MS/MS [10].

**Figure 8:**
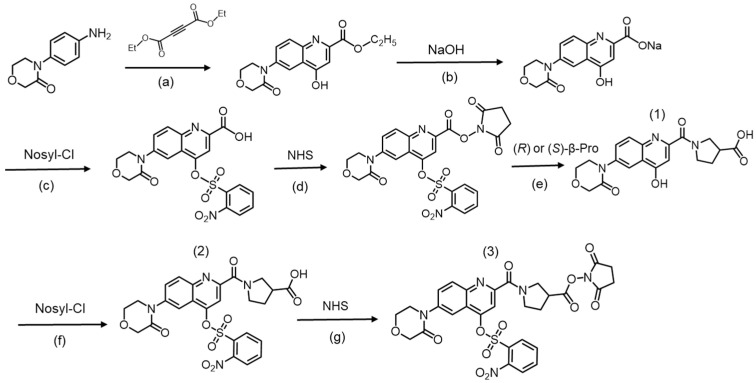
Synthetic route of Ns-MOK-β-Pro-OSu from 4-(3-oxomorpholino)aniline. (**a**) i: MeOH 80 °C 4 h, ii: Ph_2_O 220 °C 2 h; (**b**) MeOH/H_2_O r.t. 12 h, (**c**) NaHCO_3_ r.t. 2 h, (**d**) 1-(3-dimethylaminopropyl)-3-ethylcarbodiimide hydrochloride (EDC) in CH_3_CN r.t. 2 h, (**e**) DMF, NaHCO_3_ in H_2_O r.t. 2 h, (**f**) NaHCO_3_ r.t. 2 h, and (**g**) EDC in CH_3_CN r.t. 2 h; (**1**) 1-(4-hydroxy-6-(3-oxomorpholino)quinoline-2-carbonyl)pyrrolidine-3-carboxylic acid; (**2**) 1-(4-(((2-nitrophenyl)sulfonyl)oxy)-6-(3-oxomorpholino)quinoline-2-carbonyl)pyrrolidine-3-carboxylic acid; (**3**) Ns-MOK-β-Pro-OSu.

As observed in Tables S11–S15 and Figure 8, the difference in the weights of the edible parts could be attributed to the differences in the amino acid contents because significant correlations were observed between the amino acid content and the weight of the edible part. Thus far, several therapeutic applications of Tau supplements have been proposed, including the treatment of diabetes [13], hypertension [14], and heart failure [15]. A recent study reported that Tau increased the effectiveness of antipsychotics in the first episode of schizophrenia [16].

In the present study, the variations in the contents of the five amino acids were large (Table 1). The cause of these variations could be the differences in the sizes of the edible parts (Tables S16–S20). The present data suggest that consuming bivalves with large edible parts is effective for ensuring adequate intake of Tau. Moreover, a bivalve with large edible parts also contains a large amount of Glu. Excessive Glu intake may exacerbate the depressive symptoms of obese schizophrenia [17].

## 4. Materials and Methods

### 4.1. Chemicals

LCMS-grade methanol, HPLC-grade formic acid, and APDSTAG^®^ Wako Amino Acids internal standard mixture solution composed of 25 stable-isotope labeled amino acids were purchased from FUJIFILM Wako Pure Chemical Corporation (Osaka, Japan). CHCl_3_ and LCMS-grade CH_3_CN were purchased from Kanto Chemical Co., Inc. (Tokyo, Japan). The other reagents used are mentioned in Supplementary Materials, Section 1.

### 4.2. Preparation of 2,5-Dioxopyrrolidin-1-yl(4-(((2-nitrophenyl)sulfonyl)oxy)-6-(3-oxomorpholino)quinoline-2-carbonyl)pyrrolidine-3-carboxylate (Ns-MOK-β-Pro-OSu)

Ns-MOK-β-Pro-OSu was synthesized in our laboratory according to the synthetic route shown in Figure 8. The preparation of the intermediate compounds from 4-(3-oxomorpholino)aniline to yield Ns-MOK-β-Pro-OSu is described in Supplementary Materials, Section 5. The structure of the synthesized compound was confirmed through nuclear magnetic resonance (NMR) studies.

### 4.3. Time-Course Study on Derivatization of Amino Acids with Ns-MOK-Pro-OSu and Ns-MOK-β-Pro-OSu

Aliquots containing 5 μL of an amino acid mixture including Tau, (*S*)-glutamate, (*S*)-glutamine, (*S*)-arginine, and (*S*)-valine (each 100 µM) in phosphate-buffered saline (PBS) were added to 5 μL of 20 mM Ns-MOK-(*S*)-Pro-OSu in CH_3_CN or 20 mM Ns-MOK-(*S*)-β-Pro-OSu in CH_3_CN, and 5 μL of 10 mM DMAP in CH_3_CN. The solutions were allowed to react at room temperature for 5, 15, 30, 60, 90, and 120 min. Each reacted solution was diluted with 35 μL of 0.2% HCO_2_H in H_2_O/MeOH (1:1, *v*/*v*) and subjected to LC-TOF-MS (JMS-T100LP, JEOL Ltd., Tokyo, Japan) (Supplementary Materials, Section 2.2). The observed peak areas were plotted against the derivatization times.

### 4.4. Pre-Treatment Procedure

First, 10 μL of the amino acid mixture, 5.0 μL of APDSTAG^®^ (solution A:solution B = 65:5, *v*/*v*) as an internal standard (IS) solution, and 135 μL of CH_3_CN/MeOH (1:1, *v*/*v*) were mixed and precipitated for 30 min at 4 °C to yield proteins. Next, the mixture was centrifuged at 2500× *g* for 5 min at 4 °C and filtered with Millex^®^-LG (0.2 μm, Merck Ltd. Tokyo, Japan). The supernatant (100 μL) was evaporated under reduced pressure. After evaporation, the residue was dissolved in 10 μL of PBS, and vortexed with 10 μL of 20 mM Ns-MOK-(*S*)-β-Pro-OSu dissolved in CH_3_CN and 10 μL of 10 mM DMAP in CH_3_CN. The solution was reacted at room temperature for 30 min. After the reaction, 70 μL of 0.2% HCO_2_H in (H_2_O/MeOH (3:2, *v*/*v*)) was added. Then, a 10 μL aliquot was taken from this solution and diluted with 0.05% HCO_2_H in (H_2_O/MeOH (9:1, *v*/*v*)) (90 μL) and analyzed using ultra-high-performance liquid chromatography-high-resolution mass spectrometry (UHPLC-HRMS/MS, Vanquish UHPLC system and Q-Exactive^TM^_,_ standalone benchtop Quadrupole-Orbitrap high-resolution mass spectrometer, Thermo Fisher Scientific, Bremen, Germany) (Supplementary Materials, Sections 3.1 and 3.2). The measurement mode of Q-Exactive^TM^ was set to “Full MS-ddMS^2^” (Supplementary Materials, Section 3.3).

### 4.5. Assessment of Fragmentation Patterns of Derivatives

To optimize the fragmentation pattern of the derivatives, the normalized collision energy (NCE) during UHPLC-HRMS/MS was changed by 10% (from 10 to 90%) in the parallel reaction monitoring (PRM) mode used for the measurements (Supplementary Materials, Sections 3.1, 3.2, and 3.4). The precursor ion was a proton adduct of each derivatized amino acid.

### 4.6. Linearity and Limit of Quantification

Calibration curves were constructed by plotting the peak area corresponding to the IS peak ratio against the amino acid concentrations of 1, 2.5, 5, 10, 25, 50, and 100 µM. The limit of quantification (LOQ) of the amino acid derivatives determined using the proposed UHPLC-HRMS/MS technique was designated as the concentration at which the linearity of the calibration curve was lost. The NCEs were set at 20% for Val derivatives, 30% for Glu derivatives, 30% for Gln derivatives, 40% for Arg derivatives, and 30% for Tau derivatives (Supplementary Materials, Sections 8.1–8.10). The other parameters are described in Supplementary Materials, Sections 3.1, 3.2, and 3.4.

The peak areas of the amino acid derivatives were calculated using the fragment ion, C_6_H_4_N_1_O_5_S, corresponding to the characteristic fragment ion of Ns-MOK-(*S*)-β-Pro-OSu in the ESI (−) mode.

### 4.7. Pre-Treatment of Bivalve

Japanese littleneck clams, *Ruditapes philippinarum* (*R. philippinarum*), were collected from the intertidal and subtidal zones of the artificial tidal flats in Mangoku-ura Lagoon (Miyagi, Japan), and in the estuary of Udagawa in Matsukawa-ura Lagoon (Fukushima, Japan) in November 2020. The time when the clams were collected was not the spawning season.

After placing the clams in locally sampled seawater, the edible soft part was removed from the shell. Then, the water on the surface was wiped off, and the edible part of each clam was weighed.

Water was added to form a solution of the edible part with a concentration of 1 g/10 mL. After homogenization with a kitchen mixer, approximately 10 mL of the solution was taken and centrifuged at 3500× *g* for 15 min to obtain approximately 1 mL of supernatant. Furthermore, the supernatant was centrifuged twice at 13,200× *g* and 4 °C for 15 min to obtain a clear supernatant.

## 5. Conclusions

A novel derivatizing reagent, Ns-MOK-β-Pro-OSu, was developed for pre-column derivatization to determine amino acids containing Tau in bivalve using reversed-phase UHPLC-HR-MS/MS. Characteristic fragmentations under both polarities were observed in the MS/MS spectra. Therefore, highly selective quantification was possible using the characteristic fragmentation of Ns-MOK-β-Pro-OSu. The proposed method could detect 18 types of amino acids in 10 min. Considering diet therapy for ARMS based on the present results regarding the amino acid contents in the bivalve, it seems necessary to search for bivalves with a high Tau/Glu ratio. Thus, further studies are needed to investigate the Tau/Glu ratios in other species of bivalve.

## Figures and Tables

**Table 1:** Amino acid contents in the bivalve were determined using the proposed LC-HRMS/MS method.

Amino Acid Content per 1 g of the Edible Portion (mg)	Mean	±	sd
Tau	5.33	±	1.91
Val	0.28	±	0.10
Glu	1.16	±	0.49
Gln	0.20	±	0.12
Arg	0.66	±	0.33

## Data Availability

Research data are not shared.

## Supplementary Materials

**1. Chemicals and Reagents; 2. LC-Time of Flight (TOF)-Mass Spectrometry:** 2.1. Comparison of Retention Times of Ns-MOK-β-Pro-OSu and Ns-MOK-Pro-OSu and 2.2. Time-Course Study on Derivatization of Amino Acids with Ns-MOK-Pro-OSu and Ns-MOK-β-Pro-OSu; **3. Measurement Conditions of UHPLC-High-Resolution Mass Spectrometry (UHPLC-HRMS: Vanquish UHPLC System and Q-Exactive^TM^):** 3.1. Instrumentation, 3.2. Electrospray Ionization (ESI), 3.3. Parameters of Full MS-Data-Dependent MS^2^ (ddMS^2^) Mode, and 3.4. Parameters of Parallel Reaction Monitoring (PRM) Mode; **4. NMR Measurements**; **5. Preparation of 2,5-Dioxopyrrolidin-1-yl 1-(4-(((2-nitrophenyl)sulfonyl)oxy)-6-(3-oxomorpholino)quinoline-2-carbonyl)pyrrolidine-3-carboxylate (Ns-MOK-β-Pro-OSu):** 5.1. 1-(4-Hydroxy-6-(3-oxomorpholino)quinoline-2-carbonyl)pyrrolidine-3-carboxylic acid (1), 5.2. 1-(4-(((2-Nitrophenyl)sulfonyl)oxy)-6-(3-oxomorpholino)quinoline-2-carbonyl)pyrrolidine-3-carboxylic acid (2), and 5.3. 2,5-Dioxopyrrolidin-1-yl 1-(4-(((2-nitrophenyl)sulfonyl)oxy)-6-(3-oxomorpholino)quinoline-2-carbonyl)pyrrolidine-3-carboxylate (Ns-MOK-β-Pro-OSu) (3); **6. Optical Rotation Measurements of Ns-MOK-(*R*)- or (*S*)-Pro-OSu**; **7. Supplementary Figures:** Figure S1. Chromatograms of Ns-MOK-(*S*)-Pro-OSu (a) and Ns-MOK-(*S*)-β-Pro-OSu (b) obtained using LC-TOF-MS; Figure S2. Calibration curves of amino acid derivatives: (a) Tau, (b) Val, (c) Gln, (d) Glu, and (e) Arg; **8. Supplementary Tables:** Table S1. Main fragmentation of Val derivatives (C_30_H_31_N_5_O_11_S, 669.1740) as a function of NCE (ESI (+) mode), Table S2. Main fragmentation of Glu derivatives (C_30_H_29_N_5_O_13_S, 699.1482) as a function of NCE (ESI (+) mode), Table S3. Main fragmentation of Gln derivatives (C_30_H_30_N_6_O_12_S, 698.1642) as a function of NCE (ESI (+) mode), Table S4. Main fragmentation of Arg derivatives (C_31_H_34_N_8_O_11_S, 726.2067) as a function of NCE (ESI (+) mode), Table S5. Main fragmentation of Tau derivatives (C_27_H_27_N_5_O_12_S_2_, 677.1097) as a function of NCE (ESI (+) mode), Table S6. Main fragmentation of Val derivatives (C_30_H_31_N_5_O_11_S, 669.1740) as a function of NCE (ESI (−) mode), Table S7. Main fragmentation of Glu derivatives (C_30_H_29_N_5_O_13_S, 699.1482) as a function of NCE (ESI (−) mode), Table S8. Main fragmentation of Gln derivatives (C_30_H_30_N_6_O_12_S, 698.1642) as a function of NCE (ESI (−) mode), Table S9. Main fragmentation of Arg derivatives (C_31_H_34_N_8_O_11_S, 726.2067) as a function of NCE (ESI (−) mode), Table S10. Main fragmentation of Tau derivatives (C_27_H_27_N_5_O_12_S_2_, 677.1097) as a function of NCE (ESI (−) mode), Table S11. Precision of Tau/IS ratio, Table S12. Precision of the Val/IS ratio, Table S13. Precision of Gln/IS ratio, Table S14. Precision of Glu/IS ratio, Table S15. Precision of Arg/IS ratio, Table S16. Tau content in bivalve, Table S17. Val content in bivalve, Table S18. Gln content in bivalve, Table S19. Glu content in bivalve, and Table S20. Arg content in bivalve.

## Sample Availability

Samples of the compounds used are available from the authors.

## References

[1] Wu J.Y., Prentice H. (2010). Role of taurine in the central nervous system. J. Biomed. Sci..

[2] Ripps H., Shen W. (2012). Review: Taurine: A “very essential” amino acid. Mol. Vis..

[3] Menzie J., Pan C., Prentice H., Wu J.Y. (2014). Taurine and central nervous system disorders. Amino Acids.

[4] Uekusa S., Onozato M., Umino M., Sakamoto T., Ichiba H., Tsujino N., Funatogawa T., Tagata H., Nemoto T., Mizuno M. (2021). Increased inosine levels in drug-free individuals with at-risk mental state: A serum metabolomics study. Early Interv. Psychiatry.

[5] D’Aniello A., Nardi G., De Santis A., Vetere A., di Cosmo A., Marchelli R., Dossena A., Fisher G. (1995). Free l-amino acids and d-aspartate content in the nervous system of cephalopoda. A comparative study. Comp. Biochem. Physiol. B Biochem. Mol. Biol..

[6] Watanabe K., Konosu S. (1972). Presence of taurine in the extract of hard clam. Nippon Suisan Gakkai Shi.

[7] Mazzucco E., Gosetti F., Bobba M., Marengo E., Robotti E., Gennaro M.C. (2010). High-performance liquid chromatography-Ultraviolet detection method for the simultaneous determination of typical biogenic amines and precursor amino acids. Applications in Food Chemistry. J. Agric. Food Chem..

[8] Fuke S., Konosu S. (1991). Taste-active components in some foods: A review of Japanese research. Physiol. Behavior.

[9] Hernández F., Ibáñez M., Bade R., Bijlsma L., Sancho J.V. (2014). Investigation of pharmaceuticals and illicit drugs in waters by liquid chromatography-high-resolution mass spectrometry. TrAC Trends Anal. Chem..

[10] Wang X.Z., Wu H., Li N., Cheng Y., Wen H.M., Liu R., Chai C. (2016). Rapid determination of free amino acids, nucleosides, and nucleobases in commercial clam species harvested at different seasons in Jiangsu, China, using UFLC-MS/MS. Food Anal. Methods.

[11] Uekusa S., Onozato M., Sakamoto T., Umino M., Ichiba H., Fukushima T. (2021). Fluorimetric determination of the enantiomers of vigabatrin, an antiepileptic drug, by reversed-phase HPLC with a novel diastereomer derivatisation reagent. Biomed. Chromatogr..

[12] Santa T. (2013). Derivatization in liquid chromatography for mass spectrometric detection. Drug Discov. Ther..

[13] Franconi F., Loizzo A., Ghirlanda G., Seghieri G. (2006). Taurine supplementation and diabetes mellitus. Curr. Opin. Clin. Nutr. Metab. Care.

[14] Militante J.D., Lombardini J.B. (2002). Treatment of hypertension with oral taurine: Experimental and clinical studies. Amino Acids.

[15] Sole M.J., Jeejeebhoy K.N. (2000). Conditioned nutritional requirements and the pathogenesis and treatment of myocardial failure. Curr. Opin. Clin. Nutr. Metab. Care.

[16] O’Donnell C.P., Allott K.A., Murphy B.P., Yuen H.P., Proffitt T.M., Papas A., Moral J., Pham T., O’Regan M.K., Phassouliotis C. (2016). Adjunctive taurine in first-episode psychosis: A phase 2, double-blind, randomized, placebo-controlled study. J. Clin. Psychiatry.

[17] Kumar P., Kraal A.Z., Prawdzik A.M., Ringold A.E., Ellingrod V. (2020). Dietary glutamic acid, obesity, and depressive symptoms in patients with schizophrenia. Front. Psychiatry.

