# Supplementary material for: Development of a Derivatization Reagent with a 2-Nitrophenylsulfonyl Moiety for UHPLC-HRMS/MS and Its Application to Detect Amino Acids Including Taurine
Source: Molecules. 2021 Jun 8;26(12):3498. doi: 10.3390/molecules26123498 (PMC8227134; doi:10.3390/molecules26123498)
Supplement: Supplementary file 1 [file molecules-26-03498-s001.zip › molecules-1217367-supplementary.pdf]

Molecules

Supplementary Materials

Development of a Derivatization Reagent with a 2-Nitrophenylsulfonyl Moiety for UHPLC-HRMS/MS and Its Application to Detect Amino Acids Including Taurine

Shusuke Uekusa [1,2], Mayu Onozato [1], Maho Umino [1], Tatsuya Sakamoto [1], Hideaki Ichiba [1], Kenji Okoshi [3], Takeshi Fukushima [1*]

[1] Department of Analytical Chemistry, Faculty of Pharmaceutical Sciences, Toho University, Chiba, Japan

[2] Department of Clinical Pharmacy, Faculty of Pharmaceutical Sciences, Toho University, Chiba, Japan

[3] Department of Environmental Science, Faculty of Science, Toho University, Chiba, Japan.

Corresponding author: Takeshi Fukushima PhD

Department of Analytical Chemistry. Faculty of Pharmaceutical Sciences, Toho University

Miyama 2-2-1, Funabashi, Chiba 274-8510, Japan

Phone: +81-47-472-1504; Fax: +81-47-472-1504

E-mail: t-fukushima@phar.toho-u.ac.jp

Contents

1 Chemicals and reagents ……. Page 5

2 LC-time of flight (TOF)-mass spectrometry ……. Page 5

2.1 Comparison of retention times of Ns-MOK-β-Pro-OSu and Ns-MOK-Pro-OSu ……. Page 5

2.2 Time-course study on derivatization of amino acids with Ns-MOK-Pro-OSu and Ns-MOK-β-Pro-OSu …….. Page 6

3 Measurement conditions of UHPLC-high-resolution mass spectrometry (UHPLC-HRMS: Vanquish UHPLC system and Q-Exactive™) ……. Page 6

3.1 Instrumentation ……. Page 6

3.2 Electrospray ionization (ESI) ……. Page 7

3.3 Parameters of full MS-data dependent MS^2 ($ddMS^2$) mode ……. Page 7

3.4 Parameters of parallel reaction monitoring (PRM) mode ……. Page 8

4 NMR measurements ……. Page 8

5 Preparation of 2,5-dioxopyrrolidin-1-yl 1-(4-(((2-nitrophenyl)sulfonyl)oxy)-6-(3-oxomorpholino)quinoline-2-carbonyl)pyrrolidine-3-carboxylate (Ns-MOK-β-Pro-OSu) ……. Page 8

5.1 1-(4-hydroxy-6-(3-oxomorpholino)quinoline-2-carbonyl)pyrrolidine-3-carboxylic acid (1) ……. Page 9

5.2 1-(4-(((2-nitrophenyl)sulfonyl)oxy)-6-(3-oxomorpholino)quinoline-2-carbonyl) pyrrolidine-3-carboxylic acid (2) ……. Page 10

5.3 2,5-dioxopyrrolidin-1-yl 1-(4-(((2-nitrophenyl)sulfonyl)oxy)-6-(3-oxomorpholino)quinoline-2-carbonyl)pyrrolidine-3-carboxylate (Ns-MOK-β-Pro-OSu) (3) ……. Page 10

6 Optical rotation measurements of Ns-MOK-(*R*)- or (*S*)-Pro-OSu ……. Page 11

7 Supporting Figures

7.1 FIGURE S1 Chromatograms of Ns-MOK-(*S*)-Pro-OSu (a) and Ns-MOK-(*S*)-β-Pro-OSu (b) obtained using LC-TOF-MS ……. Page 13

7.2 FIGURE S2 Calibration curves of amino acid derivatives: (a) Tau, (b) Val, (c) Gln, (d) Glu, and (e) Arg ……. Page 14

8 Supporting Tables

8.1 Table S1 Main fragmentation of Val derivatives ($C_{30}H_{31}N_5O_{11}S$, 669.1740) as a function of NCE (ESI (+) mode) ……. Page 15

8.2 Table S2 Main fragmentation of Glu derivatives ($C_{30}H_{29}N_5O_{13}S$, 699.1482) as a function of NCE (ESI (+) mode) ……. Page 15

8.3 Table S3 Main fragmentation of Gln derivatives ($C_{30}H_{30}N_6O_{12}S$, 698.1642) as a function of NCE (ESI (+) mode) ……. Page 16

8.4 Table S4 Main fragmentation of Arg derivatives ($C_{31}H_{34}N_8O_{11}S$, 726.2067) as a function of NCE (ESI (+) mode) ……. Page 16

8.5 Table S5 Main fragmentation of Tau derivatives ($C_{27}H_{27}N_5O_{12}S_2$, 677.1097) as a function of NCE (ESI (+) mode) ……. Page 17

8.6 Table S6 Main fragmentation of Val derivatives ($C_{30}H_{31}N_5O_{11}S$,

669.1740) as a function of NCE (ESI (-) mode) ……. Page 17

8.7 Table S7 Main fragmentation of Glu derivatives ($C_{30}H_{29}N_5O_{13}S$, 699.1482) as a function of NCE (ESI (-) mode) ……. Page 18

8.8 Table S8 Main fragmentation of Gln derivatives ($C_{30}H_{30}N_6O_{12}S$, 698.1642) as a function of NCE (ESI (-) mode) ……. Page 18

8.9 Table S9 Main fragmentation of Arg derivatives ($C_{31}H_{34}N_8O_{11}S$, 726.2067) as a function of NCE (ESI (-) mode) ……. Page 19

8.10 Table S10 Main fragmentation of Tau derivatives ($C_{27}H_{27}N_5O_{12}S_2$, 677.1097) as a function of NCE (ESI (-) mode) ……. Page 19

8.11 Table S11 Precision of Tau/IS ratio ……. Page 20

8.12 Table S12 Precision of Val/IS ratio ……. Page 20

8.13 Table S13 Precision of Gln/IS ratio ……. Page 20

8.14 Table S14 Precision of Glu/IS ratio ……. Page 20

8.15 Table S15 Precision of Arg/IS ratio ……. Page 21

8.16 Table S16 Tau content in bivalve ……. Page 21

8.17 Table S17 Val content in bivalve ……. Page 22

8.18 Table S18 Gln content in bivalve ……. Page 23

8.19 Table S19 Glu content in bivalve ……. Page 24

8.20 Table S20 Arg content in bivalve ……. Page 25

1 Chemicals and reagents

(*S*)- and (*R*)-Pyrrolidine-3-carboxylic acid (β-proline) were purchased from Sigma-Aldrich Co., Ltd. (St. Louis, MO, USA). Arginine (Arg), glutamine (Gln), glutamic acid (Glu), taurine (Tau), valine (Val), 4-(3-oxomorpholino)aniline, and 4-dimethylaminopyridine (DMAP) were purchased from Tokyo Chemical Industries Co., Ltd. (Tokyo, Japan). 1-(3-dimethylaminopropyl)-3-ethylcarbodiimide hydrochloride (EDC), diphenyl ether, diethyl acetylenedicarboxylate, LC-MS-grade CH_3OH, HPLC-grade acetic acid, formic acid, and APDSTAG® Wako Amino Acids Internal Standard Mixture Solution were purchased from FUJIFILM Wako Pure Chemical Corporation (Osaka, Japan). LCMS-grade acetonitrile and chloroform were purchased from Kanto Chemical Co., Inc. (Tokyo, Japan).

2 LC-time of flight (TOF)-mass spectrometry

The LC-TOF-MS equipment (JMS-T100LP, JEOL, Tokyo, Japan) with an intelligent HPLC system, M1000 (Agilent Technologies, Inc., Santa Clara, CA), was used for confirming each structure.

2.1 Comparison of retention times of Ns-MOK-β-Pro-OSu and Ns-MOK-Pro-OSu

The retention times of Ns-MOK-β-Pro-OSu and Ns-MOK-Pro-OSu were determined using the LC-TOF-MS equipment. The separation column used was InertSustain® C18 (3 μm, 2.1×100 mm) (GL Sciences Inc., Tokyo, Japan) maintained at 40°C. The mobile phases were 0.05% formic acid in [H_2O/ CH_3CN (4/1, v/v)] (A) and 0.05% formic acid in [H_2O/ CH_3CN (1/4, v/v)] (B), and eluted at 0.2 mL/min according to the following time program: 0–5 min; B% = 30, 5–40 min; B% = from 30 to 100, 40-

48 min; B% = 100, 48.0-48.1 min; B% = from 100 to 30, 48.1-56 min; B% = 30. In the MS system, the ion source was electron spray ionization (ESI) (+), and the desolvation chamber and orifice temperatures were 250 and 80°C, respectively. The voltages of orifices 1 and 2 were 50 and 5 V, respectively, and the peak-to-peak and needle voltages were 500 and 2500 V, respectively.

The prepared samples were dissolved in CH_3CN (10 mM) and diluted 10-fold with a mobile phase (A). A 10 μL aliquot of the final solution was injected into the LC-TOF-MS equipment.

2.2 Time-course study on derivatization of amino acids with Ns-MOK-Pro-OSu and Ns-MOK-β-Pro-OSu.

LC-TOF-MS was used for the time-course study of the derivatization of Tau, Arg, Glu, and Gln with Ns-MOK-β-Pro-OSu and Ns-MOK-Pro-OSu. The separation column used was InertSustain® C18 (3 μm, 2.1×100 mm) (GL Sciences Inc., Tokyo, Japan) maintained at 60°C. The mobile phases were 0.05% formic acid in [H_2O/MeOH (9/1, v/v)] (A) and 0.05% formic acid in [H_2O/MeOH (1/9, v/v)] (B), and eluted at 0.2 mL/min according to the following time program: 0–40 min; B% = 30–100, 40–50 min; B% = 100, 50-50.01 min; B% = 100–30, 50.01-60 min; B% = 30. The TOF-MS system used was the same as that described in **Section 2.1**. A 10 μL aliquot of the final solution was injected into the LC-TOF/MS equipment.

3 Measurement conditions of UHPLC-high-resolution mass spectrometry (UHPLC-HRMS: Vanquish UHPLC system and Q-Exactive™)

3.1 Instrumentation

The instrument was calibrated before measurement using a ready-to-use calibration mixture. Briefly, a Vanquish UHPLC system equipped with an Acquity HSS T3 C18 column (1.8 µm, 75 × 2.1 mm) (Waters Corporation, Milford, MA) was used for chromatographic separation. A binary solvent system comprising mobile phases A (0.05% in [H_2O/ MeOH (9/1, v/v)]) and B (0.05% in [H_2O/ MeOH (1/9, v/v)]) was used at a constant flow rate of 0.4 mL/min at 45°C. The gradient elution was then performed using the following solvent proportions: A: 0‑5 min; from 30 to 35, 5.0–5.1 min; 35% to 65%, 5.1–10 min; 65%, 10.0–10.1 min; from 65% to 100%, 10.1–13.0 min; 100%, 13.0–13.1 min; from 100 to 30%, 13.1-16.0 min; B% = 30%. Then, 5.0 µL of the filtered sample solution described above was injected into the UHPLC system.

Detection was performed on a Q-Exactive™ standalone bench-top Quadrupole-Orbitrap high-resolution mass spectrometer, which was preceded by heated electrospray ionization (HESI-II source).

3.2 Electrospray ionization (ESI)

During ESI, the spray voltages were -2.50 kV and 3.50 kV for the negative and positive ion modes, respectively. The following conditions were common in both ionization modes: sheath gas, auxiliary gas, sweep gas flow rates of 50, 13, and 3 arbitrary units, capillary and auxiliary gas heater temperatures of 263 and 425°C, respectively, and S-lens RF level of 50.0.

3.3 Parameters of full MS-data dependent MS^2 ($ddMS^2$) mode

The parameters for “Full MS-ddMS^2” were as follows: mass range (*m/z*) from 350 to 2,000, resolution of 17,500, AGC target at 100,000, normalized collision energy

(NCE) stepped at 15, 30, and 45%, loop count at 5, isolation window at 1.6 *m/z*, minimum AGC target at 5000, and dynamic exclusion at 15.0 s

3.4 Parameters of parallel reaction monitoring (PRM) mode

The parameters for "PRM" were as follows: inclusion list with $C_{30}H_{31}N_5O_{11}S$ (Val and Val-$^{13}C_3$,^{15}N derivative), $C_{30}H_{29}N_5O_{13}S$ (Glu and Glu-$^{13}C_3$,^{15}N derivative), $C_{30}H_{30}N_6O_{12}S$ (Gln and Gln-$^{13}C_3$,$^{15}N_2$ derivative), $C_{31}H_{34}N_8O_{11}S$ (Arg and Arg-$^{15}N_4$ derivative), and $C_{27}H_{27}N_5O_{12}S_2$ (Tau and Tau-$^{13}C_2$ derivative), resolution at 17,500, AGC target at 20,000, maximum IT at 100 ms, and isolation window at 0.5 *m/z*

4 NMR measurements

The prepared compounds were dissolved in DMSO-D_6 or $CDCl_3$, and the proton NMR (^{1}H-NMR) spectra of the prepared compounds were recorded on a JMS-ECS 400 spectrometer (400 MHz) (JEOL Ltd., Tokyo, Japan) using tetramethylsilane (TMS) as the internal standard. Chemical shifts are expressed as parts per million relative to TMS.

5 Preparation of 2,5-dioxopyrrolidin-1-yl 1-(4-(((2-nitrophenyl)sulfonyl)oxy)-6-(3-oxomorpholino)quinoline-2-carbonyl)pyrrolidine-3-carboxylate (Ns-MOK-β-Pro-OSu)

We synthesized 2,5-dioxopyrrolidin-1-yl 4-(((2-nitrophenyl)sulfonyl)oxy)-6-(3-oxomorpholino)quinoline-2-carboxylate using the method reported earlier [1]. The reaction scheme was shown in **FIGURE 8**.

5.1 1-(4-hydroxy-6-(3-oxomorpholino)quinoline-2-carbonyl)pyrrolidine-3-carboxylic acid (1)

Crude 2,5-dioxopyrrolidin-1-yl 4-(((2-nitrophenyl)sulfonyl)oxy)-6-(3-oxomorpholino)quinoline-2-carboxylate (494.5 mg, 0.87 mmol) was dissolved in dehydrated CH_3CN (5 mL), and then DMAP (210.1 mg, 1.7 mmol, 2.0 eq.) in CH_3CN (2 mL) and (*R*)-(-)- or (*S*)-(+)-pyrolidine-3-carboxylic (200.1 mg, 1.7 mmol, 2.0 eq. in H_2O (2 mL) were added. The solution was stirred at room temperature for 2 h. The reaction mixture was evaporated under reduced pressure. The residue was dissolved in 0.2% CH_3CO_2H in H_2O/CH_3CN (95:5,10 mL). The solution was acidified to pH 3‑4 using 6 M HCl. The mixture was purified using preparative reversed-phase column chromatography (Yamazen Corporation ULTRA PACK, ODS-SM, 50 µm, 120 Å, Size A, 11×300 mm) with a mobile phase [(0.2% CH_3CO_2H in H_2O/CH_3CN (95:5)] flow using a pump module (C601 BÜCHI Labortechnik GmbH, Essen, Germany) at a flow rate of 0.4 mL/min. The target fraction was collected and evaporated under reduced pressure. Light yellow crystals of 1-(4-hydroxy-6-(3-oxomorpholino)quinoline-2-carbonyl)pyrrolidine-3-carboxylic acid [1] were obtained (124.3 mg, 0.32 mmol, 37.2%).

$C_{19}H_{19}N_3O_6$, theoretical exact mass: 385.1274, experimental $(M+H)^+$: 386.2229

^{1}H-NMR (400 MHz, Methanol-D_4) δ 8.08–8.25 (1H, Ar*H*), 7.60–7.88 (2H, Ar*H*), 6.35–6.55 (1H, Ar*H*), 4.23–4.35 (2H, $COCH_2O$), 3.95–4.19 (2H, NCH_2CH_2O), 3.81–3.96 (2H, NCH_2CH_2O), 3.58–3.79 (2H, NCH_2CH), 3.04–3.23 (1H, $(CH_2)_2CHCO$), 2.11–2.39 (2H, CH_2CH_2N), and 1.90–2.02 (2H, CH_2CH_2CH)

5.2 1-(4-(((2-nitrophenyl)sulfonyl)oxy)-6-(3-oxomorpholino)quinoline-2-carbonyl)pyrrolidine-3-carboxylic acid (2)

Subsequently, (1) was dissolved in H_2O (10 mL) and $NaHCO_3$ (271.3 mg, 3.2 mmol, 10.0 eq. It was then cooled over ice and a solution of 4-nitrobenzenesulfonyl chloride (282.9, 1.3 mmol, 4.0 eq. in acetone (5 mL) was added dropwise, and the mixture was stirred at room temperature for 2 h. The reaction mixture was then transferred to saturated $NaHCO_3$ (100 mL) and AcOEt (100 mL) in a separating funnel. The aqueous layer was acidified to pH 3 using 6 M HCl. The mixture was extracted with $CHCl_3$ (100 mL ×3) in a separating funnel. The organic layer was dried over Na_2SO_4 after washing with brine (100 mL). The solvent was evaporated under reduced pressure. Crude 1-(4-(((2-nitrophenyl)sulfonyl)oxy)-6-(3-oxomorpholino)quinoline-2-carbonyl)pyrrolidine-3-carboxylic acid (2) was obtained as white/light yellow crystals (146.1 mg, 0.26 mmol, 79.3%).

$C_{25}H_{22}N_4O_{10}S$, theoretical exact mass: 570.1057, experimental $(M+H)^+$: 571.1267

^{1}H-NMR (400 MHz, Methanol-D_4) δ 7.60-8.31 (8H, Ar*H*), 4.27–4.33 (2H, $COCH_2O$), 3.64–4.19 (8H, NCH_2CH_2O, NCH_2CH_2O, NCH_2CH, CH_2CH_2N), 3.15–3.26 (1H, $(CH_2)_2CHCO$), and 2.11–2.39 (2H, CH_2CH_2CH)

5.3 2,5-dioxopyrrolidin-1-yl 1-(4-(((2-nitrophenyl)sulfonyl)oxy)-6-(3-oxomorpholino)quinoline-2-carbonyl)pyrrolidine-3-carboxylate (Ns-MOK-β-Pro-OSu) (3)

First, (2) was dissolved in dehydrated $CHCl_3$ (10 mL); NHS (115.0 mg, 1.0 mmol, 4.0 equiv.) and EDC (191.7 mg, 1.0 mmol, 4.0 equiv.) in dehydrated CH_3CN (10 mL) were added. The solution was stirred at room temperature for 2 h. The reaction mixture was extracted with $CHCl_3$ (100 mL) and the organic layer was washed with

saturated $NaHCO_3$ (100 mL), followed by 0.2 M HCl (100 mL) and brine (100 mL) in a separating funnel.

The organic layer was dried over Na_2SO_4, and the solvent was evaporated under reduced pressure. The residue was dissolved in H_2O/CH_3CN (2:1,5 mL). The mixture was purified using preparative reversed-phase column chromatography (Yamazen Corporation ULTRA PACK, ODS-SM, 50 µm, 120 Å, Size A, 11×300 mm) with a mobile phase (H_2O/CH_3CN (2:1)) flow using a pump module (C601, BÜCHI Labortechnik GmbH, Essen, Germany) at a flow rate of 0.6 mL/min. The target fraction was collected and extracted using $CHCl_3$ (100 mL). The organic layer was washed with saturated $NaHCO_3$ (100 mL), followed by 0.2 M HCl (100 mL) and brine (100 mL) in a separating funnel. The organic layer was dried over Na_2SO_4, and the solvent was evaporated under reduced pressure. White crystals of 2,5-dioxopyrrolidin-1-yl 1-(4-(((2-nitrophenyl)sulfonyl)oxy)-6-(3-oxomorpholino)quinoline-2-carbonyl)pyrrolidine-3-carboxylate (Ns-MOK-β-Pro-OSu) (3) were obtained (57.0 mg, 0.085 mmol, 32.7%). $C_{29}H_{25}N_5O_{12}S$, theoretical exact mass: 667.1220, experimental $(M+H)^+$: 668.0740 ^{1}H-NMR (400 MHz, Chloroform-D) δ 8.08–8.17 (3H, Ar*H*), 7.94–8.03 (2H, Ar*H*), 7.84–7.91 (2H, Ar*H*), 7.72–7.82 (1H, Ar*H*), 4.32–4.49 (3H, $COCH_2O$, NCH_2CH), 4.04–4.23 (4H, NCH_2CH_2O), 3.91–3.98 (2H, CH_2CH_2N), 3.44–3.55 (1H, NCH_2CH), 2.72–3.00 (5H, $COCH_2CH_2CO$, $(CH_2)_2CHCO$), and 2.27–2.67 (2H, CH_2CH_2CH)

6 Optical rotation measurements of Ns-MOK-(*R*)- or (*S*)-Pro-OSu

The optical rotation of each optically active isomer of Ns-MOK-(*R*)- or (*S*)-β-Pro-OSu was measured using a polarimeter (P-2200, Jasco Corporation, Tokyo, Japan) with a 100-mm-long glass cell. The (*R*)- and (*S*)-enantiomers were dissolved in $CHCl_3$ at concentrations of 0.445 and 0.650% (w/v), respectively. The specific rotations using

sodium D-line (589 nm) irradiation at room temperature were measured.

Their specific rotations $[\alpha]_D$ showed opposite polarities (– 27.7° and + 13.9°, respectively).

7 Supporting Figures

7.1

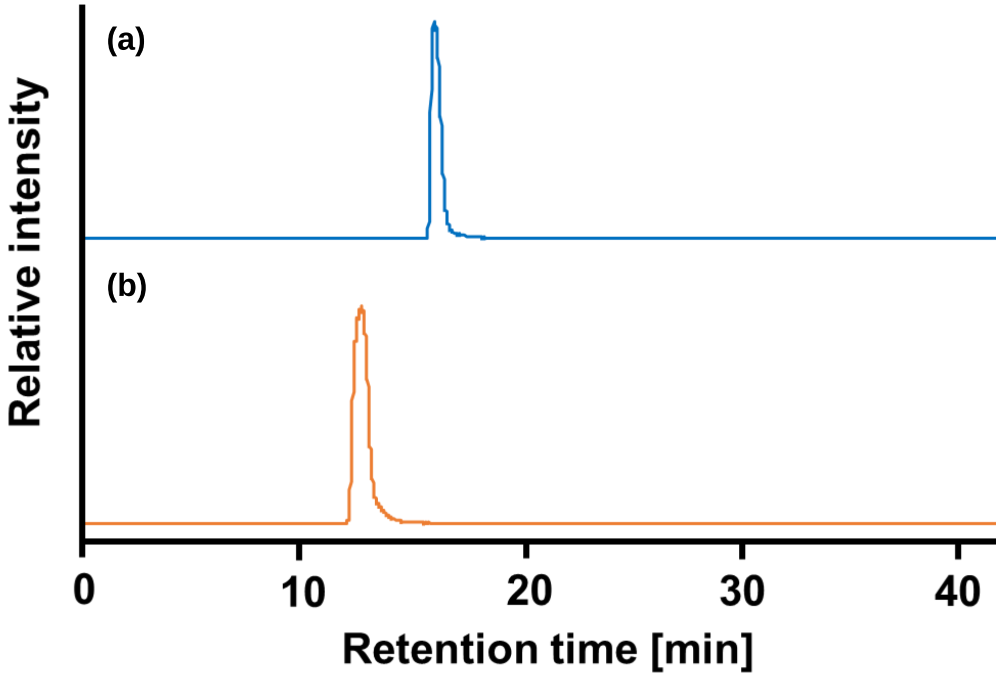

FIGURE S1 **Chromatograms of Ns-MOK-(*S*)-Pro-OSu (a) and Ns-MOK-(*S*)-β-Pro-OSu (b) obtained using LC-TOF-MS.**

7.2

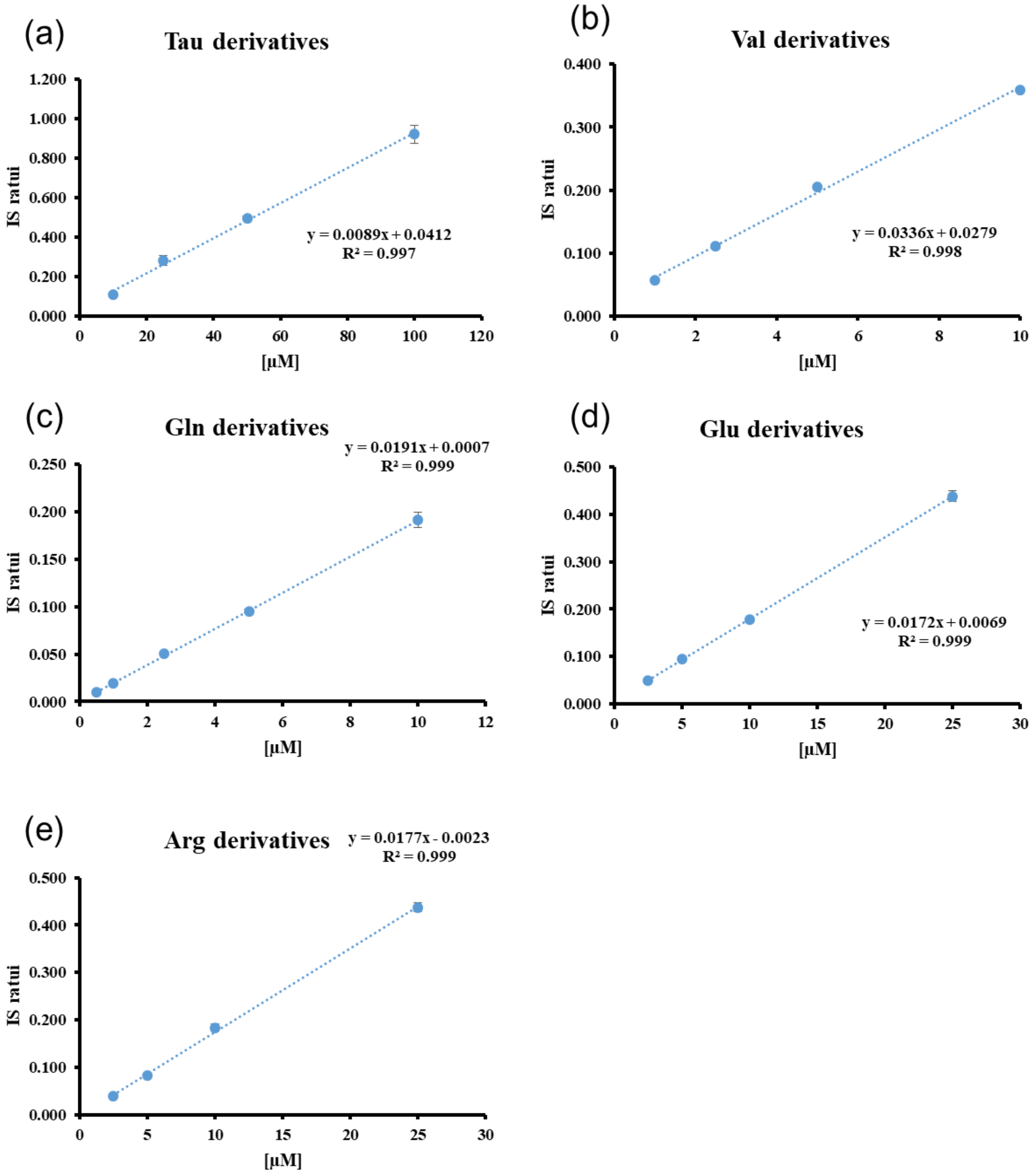

FIGURE S2 **Calibration curves of amino acid derivatives: (a) Tau, (b) Val, (c) Gln, (d) Glu, and (e) Arg**

8 Supporting Tables

8.1

Table S1 Main fragmentation of Val derivatives ($C_{30}H_{31}N_5O_{11}S$, 669.1740) as a function of NCE (ESI (+) mode)

Fragment ion	NCE
482.1424	10-20
259.0711	30-60
213.1232	40-90
96.0443	40-90
70.0651	40-90

8.2

Table S2 Main fragmentation of Glu derivatives ($C_{30}H_{29}N_5O_{13}S$, 699.1482) as a function of NCE (ESI (+) mode)

Fragment ion	NCE
312.1347	30
259.0711	40-60
243.0972	30
159.9981	30
96.0444	60-90
80.9480	30
68.0494	70-90

8.3

Table S3 Main fragmentation of Gln derivatives ($C_{30}H_{30}N_6O_{12}S$, 698.1642) as a function of NCE (ESI (+) mode)

Fragment ion	NCE
259.0716	50-60
242.1134	30
147.0765	40
96.0445	40-90

8.4

Table S4 Main fragmentation of Arg derivatives ($C_{31}H_{34}N_8O_{11}S$, 726.2067) as a function of NCE (ESI (+) mode)

Fragment ion	NCE
270.1558	30-40
261.0869	60-80
201.0981	40-50
96.0444	50-90
70.0652	50-90

8.5

Table S5 Main fragmentation of Tau derivatives ($C_{27}H_{27}N_5O_{12}S_2$, 677.1097) as a function of NCE (ESI (+) mode)

Fragment ion	NCE
312.1347	30
259.0711	30-80
221.0591	30-40
185.9852	40
96.0444	50-90
70.0651	60-90
68.0494	70-90

8.6

Table S6 Main fragmentation of Val derivatives ($C_{30}H_{31}N_5O_{11}S$, 669.1740) as a function of NCE (ESI (-) mode)

Fragment ion	NCE
243.0778	60-70
201.9817	10-50
185.0718	80
96.9698	10-80
79.9592	10-80

8.7

Table S7 Main fragmentation of Glu derivatives ($C_{30}H_{29}N_5O_{13}S$, 699.1482) as a function of NCE (ESI (-) mode)

Fragment ion	NCE
243.0775	50-60
201.9822	10-50
185.0719	80
163.8407	10
129.8716	30-70
92.9272	10
78.9573	60-90

8.8

Table S8 Main fragmentation of Gln derivatives ($C_{30}H_{30}N_6O_{12}S$, 698.1642) as a function of NCE (ESI (-) mode)

Fragment ion	NCE (%)
291.8399	10
259.0716	50-60
201.9815	20-30
147.0767	40
127.8736	30
96.0445	40-60
79.9574	80-90

8.9

Table S9 Main fragmentation of Arg derivatives ($C_{31}H_{34}N_8O_{11}S$, 726.2067) as a function of NCE (ESI (-) mode)

Fragment ion	NCE
391.2853	10
268.8007	20
243.0774	60-70
201.9810	20-40
185.0726	80
92.9283	10-90
78.9590	80-90

8.10

Table S10 Main fragmentation of Tau derivatives ($C_{27}H_{27}N_5O_{12}S_2$, 677.1097) as a function of NCE (ESI (-) mode)

Fragment ion	NCE
219.0445	30-60
201.9812	20
163.8404	10
149.9865	30
124.0073	60-70
92.9281	10
79.9574	20-90

8.11

Table S11 Precision of Tau/IS ratio

Tau (μM)	100	50	25	10
mean	0.921	0.496	0.281	0.109
sd	0.046	0.011	0.025	0.008
CV%	4.94143	2.12266	8.75605	7.29962

8.12

Table S12 Precision of Val/IS ratio

Val (μM)	10	5	2.5	1
mean	0.360	0.205	0.111	0.057
sd	0.002	0.002	0.004	0.001
CV%	0.680	1.047	3.524	1.945

8.13

Table S13 Precision of Gln/IS ratio

Gln (μM)	10	5	2.5	1	0.5
mean	0.191	0.095	0.051	0.019	0.010
sd	0.008	0.002	0.002	0.001	2.3×10^{-4}
CV%	4.113	2.122	4.757	5.781	2.430

8.14

Table S14 Precision of Glu/IS ratio

Glu (μM)	25	10	5	2.5
mean	0.438	0.177	0.095	0.050
sd	0.012	0.005	0.001	0.001
CV%	2.701	2.710	0.635	2.445

8.15

Table S15 Precision of Arg/IS ratio

**Arg (μM)**	**25**	**10**	**5**	**2.5**
**mean**	**0.437**	**0.184**	**0.083**	**0.039**
**sd**	**0.009**	**0.007**	**0.002**	**0.003**
**CV%**	**2.121**	**4.046**	**2.281**	**7.331**

8.16

Table S16 Tau content in bivalve

Sample No.	Tau IS ratio		Tau Conc.(mM)	Weight of edible portion (g)	Tau content per gram of the edible portion (mg)
	mean	sd			
No. 1	0.318	0.004	3.11	1.29	3.90
No. 2	0.476	0.017	4.88	2.69	6.11
No. 3	0.402	0.026	4.06	1.69	5.08
No. 4	0.355	0.005	3.52	1.41	4.41
No. 5	0.342	0.008	3.39	1.75	4.24
No. 6	0.419	0.009	4.25	2.34	5.32
No. 7	0.553	0.013	5.75	2.58	7.20
No. 8	0.352	0.011	3.49	2.64	4.37
No. 9	0.600	0.016	6.28	2.57	7.86
No. 10	0.781	0.026	8.31	3.05	10.40
No. 11	0.326	0.022	3.21	1.66	4.01
No. 12	0.443	0.009	4.51	1.17	5.65
No. 13	0.292	0.014	2.82	1.96	3.53
No. 14	0.274	0.011	2.62	1.36	3.28
No. 15	0.372	0.011	3.72	1.46	4.66

No. 1–5: Japanese littleneck clam from the intertidal zone in Mangoku-ura Laggon, No. 6–10: Japanese littleneck clam from the subtidal zone in Mangoku-ura Lagoon, and No. 11–15: Japanese littleneck clam from estuary of Udagawa in Matsukawa-ura Lagoon

8.17

Table S17 Val content in bivalve

Sample No.	Val IS ratio		Val Conc.(mM)	Weight of edible portion (g)	Val content per gram of the edible portion (mg)
	mean	sd			
No. 1	0.075	0.002	0.14	1.29	0.16
No. 2	0.113	0.001	0.25	2.69	0.30
No. 3	0.097	0.003	0.21	1.69	0.24
No. 4	0.081	0.003	0.16	1.41	0.19
No. 5	0.088	0.002	0.18	1.75	0.21
No. 6	0.114	0.002	0.26	2.34	0.30
No. 7	0.145	0.003	0.35	2.58	0.41
No. 8	0.090	0.007	0.19	2.64	0.22
No. 9	0.136	0.001	0.32	2.57	0.38
No. 10	0.175	0.004	0.44	3.05	0.51
No. 11	0.093	0.005	0.19	1.66	0.23
No. 12	0.120	0.004	0.28	1.17	0.32
No. 13	0.094	0.003	0.20	1.96	0.23
No. 14	0.080	0.001	0.16	1.36	0.18
No. 15	0.109	0.005	0.24	1.46	0.28

No. 1–5: Japanese littleneck clam from the intertidal zone in Mangoku-ura Lagoon, No. 6–10: Japanese littleneck clam from the subtidal zone in Mangoku-ura Lagoon, and No. 11–15: Japanese littleneck clam from estuary of Udagawa in Matsukawa-ura Lagoon

8.18

Table S18 Gln content in bivalve

Sample No.	Gln IS ratio		Gln Conc.(mM)	Weight of edible portion (g)	Gln content per gram of the edible portion (mg)
	mean	sd			
No. 1	0.021	0.001	0.11	1.29	0.15
No. 2	0.051	0.011	0.26	2.69	0.39
No. 3	0.021	0.001	0.11	1.69	0.15
No. 4	0.020	0.001	0.10	1.41	0.15
No. 5	0.024	0.003	0.12	1.75	0.18
No. 6	0.027	0.001	0.14	2.34	0.20
No. 7	0.045	0.002	0.23	2.58	0.34
No. 8	0.019	0.002	0.09	2.64	0.14
No. 9	0.046	0.001	0.24	2.57	0.35
No. 10	0.060	0.006	0.31	3.05	0.46
No. 11	0.011	0.000	0.05	1.66	0.08
No. 12	0.010	0.002	0.05	1.17	0.08
No. 13	0.013	0.000	0.06	1.96	0.09
No. 14	0.017	0.001	0.09	1.36	0.13
No. 15	0.016	0.002	0.08	1.46	0.12

No. 1–5: Japanese littleneck clam from the intertidal zone in Mangoku-ura Lagoon, No. 6–10: Japanese littleneck clam from the subtidal zone in Mangoku-ura Lagoon, and No. 11–15: Japanese littleneck clam from estuary of Udagawa in Matsukawa-ura Lagoon

8.19

Table S19 Glu content in bivalve

Sample no.	Glu IS ratio		Glu Conc.(mM)	Weight of edible portion (g)	Glu content per gram of the edible portion (mg)
	mean	sd			
No. 1	0.079	0.006	0.42	1.29	0.62
No. 2	0.151	0.002	0.84	2.69	1.24
No. 3	0.110	0.005	0.60	1.69	0.88
No. 4	0.107	0.005	0.58	1.41	0.86
No. 5	0.099	0.004	0.54	1.75	0.79
No. 6	0.151	0.020	0.84	2.34	1.23
No. 7	0.188	0.004	1.05	2.58	1.55
No. 8	0.134	0.003	0.74	2.64	1.09
No. 9	0.204	0.007	1.15	2.57	1.69
No. 10	0.296	0.010	1.68	3.05	2.48
No. 11	0.110	0.008	0.60	1.66	0.88
No. 12	0.174	0.020	0.97	1.17	1.43
No. 13	0.096	0.009	0.52	1.96	0.76
No. 14	0.085	0.004	0.46	1.36	0.67
No. 15	0.149	0.002	0.82	1.46	1.21

No. 1–5: Japanese littleneck clam from the intertidal zone in Mangoku-ura, No. 6–10: Japanese littleneck clam from the subtidal zone in Mangoku-ura, and No. 11–15: Japanese littleneck clam from estuary of Udagawa in Matsukawa-ura Lagoon

8.20

Table S20 Arg content in bivalve

Sample No.	Arg IS ratio		Arg Conc.(mM)	Weight of edible portion (g)	Arg content per gram of the edible portion (mg)
	mean	sd			
No. 1	0.044	0.002	0.26	1.29	0.45
No. 2	0.089	0.006	0.51	2.69	0.90
No. 3	0.038	0.006	0.23	1.69	0.40
No. 4	0.047	0.002	0.28	1.41	0.49
No. 5	0.056	0.006	0.33	1.75	0.58
No. 6	0.065	0.005	0.38	2.34	0.66
No. 7	0.110	0.003	0.64	2.58	1.11
No. 8	0.059	0.004	0.35	2.64	0.60
No. 9	0.102	0.006	0.59	2.57	1.03
No. 10	0.148	0.018	0.85	3.05	1.47
No. 11	0.038*	0.000	0.23	1.66	0.40
No. 12	0.059	0.006	0.35	1.17	0.60
No. 13	0.031*	0.005	0.19	1.96	0.32
No. 14	0.031*	0.002	0.19	1.36	0.33
No. 15	0.050	0.010	0.30	1.46	0.51

No. 1–5: Japanese littleneck clam from the intertidal zone in Mangoku-ura, No. 6–10: Japanese littleneck clam from the subtidal zone in Mangoku-ura, and No. 11–15: Japanese littleneck clam from estuary of Udagawa in Matsukawa-ura Lagoon

***** Values below the lower limit of the calibration curve. They are reference values, but they are calculated by extrapolating the calibration curve.

References

1. Uekusa, S.; Onozato, M.; Sakamoto, T.; Umino, M.; Ichiba, H.; Fukushima, T. Fluorimetric Determination of the Enantiomers of Vigabatrin, an Antiepileptic Drug, by Reversed-Phase HPLC with a Novel Diastereomer Derivatisation Reagent. *Biomed. Chromatogr.* **2021**, *35*, e5060, doi:10.1002/bmc.5060.
